# The Use of Fractional Flow Reserve for Physiological Assessment of Indeterminate Lesions in Peripheral Artery Disease

**DOI:** 10.7759/cureus.4445

**Published:** 2019-04-12

**Authors:** Muhammad A Mangi, Rehan Kahloon, Ahmed Elzanaty, FNU Zafrullah, Ehab Eltahawy

**Affiliations:** 1 Cardiology, University of Toledo Medical Center, Toledo, USA; 2 Interventional Cardiovascular Medicine, University of Toledo Medical Center, Toledo, USA; 3 Internal Medicine, University of Toledo Medical Center, Toledo, USA; 4 Internal Medicine, Steward Carney Hospital, Tufts University School of Medicine, Boston, USA

**Keywords:** hyperemia, fractional flow reserve, ankle brachial index, doppler ultrasound

## Abstract

Peripheral artery disease (PAD) is a prevalent disorder in the United States, associated with significant morbidity and mortality. Fractional Flow Reserve (FFR) is a physiological test used to assess the hemodynamic significance of intermediate lesions on conventional angiography. It is well studied in coronary artery disease and is as an important tool to guide decisions regarding revascularization in a significant percentage of patients with intermediate lesions. As compared to coronary FFR, the use of FFR in peripheral artery disease (PFFR) is much less prevalent. Overall data regarding the use of the PFFR is sparse. There are limited studies that have shown the correlation of PFFR with non-invasive testing including ankle-brachial index (ABI) and Doppler Imaging. Unlike coronary FFR, the optimal pharmaceutical agents and doses to induce maximal hyperemia in the peripheral vascular bed are also not well established. Moreover, there are no established standardized procedural protocols for measuring PFFR. Various studies have employed varying techniques, hyperemic agents and doses.

The aim of this literature review is to summarize the current evidence on PFFR, the correlation with noninvasive studies used in PAD and to increase awareness of the potential role of the PFFR in peripheral interventions.

## Introduction and background

Peripheral artery disease (PAD) is characterized by progressive narrowing of the peripheral vascular bed. PAD is a prevalent disorder, affecting 14 million per year of the United States population [1]. PAD causes a significant effect on the quality of life, including claudication, limited mobility, loss of tissue and limb loss. Endovascular therapy (EVT) is an important treatment option for patients with symptomatic peripheral artery disease despite a supervised exercise program and maximally tolerated medical therapy.

Fractional Flow Reserve (FFR) is an important tool to identify the hemodynamic significance of vascular lesions in the intermediate stenosis category. FFR is well investigated in coronary artery disease, and commonly used in daily practice [2]. Coronary FFR (CFFR) has been shown to predict adverse events in decisions regarding coronary intervention [3,4]. However, there is no strong literature regarding the use of FFR in peripheral interventions (PFFR).

The aim of this literature review is to present the current evidence on PFFR, the safety and role of the PFFR in peripheral interventions and its correlation to non-invasive imaging modalities.

## Review

Fractional flow reserve

FFR is defined as a ratio of maximum achievable blood flow through stenosis to the maximum achievable blood flow through the same vessel in hypothetically absent stenosis. It is calculated by a ratio of pressure measured distal (Pd) to the stenosis to the pressure measured proximal (Pa) to the stenosis. The Pd/Pa ratio is 1 in a normal healthy vessel free of stenosis.

Conventional two-dimensional vascular angiography can underestimate or overestimate lesion severity. Several factors other than the percentage of luminal narrowing have been identified in causing ischemia, including the length of stenosis, the amount and viability of the tissue supplied by affected vessel, and the presence or absence of collaterals and bypass grafts to the affected vessel.

FFR is measured by passing a specialized wire with a distal pressure transducer distal to the lesion. After the wire crosses distally to the lesion, maximal hyperemia is achieved by giving a vasodilator (Adenosine or papaverine or nitroglycerine). Thereafter, FFR is calculated as the ratio of the pressure distal to pressure proximal to the lesion (Pd/Pa). FFR measured by a dedicated pressure-wire is more accurate than measuring with a catheter [5]. As compared to a pressure wire, a guiding catheter occupies additional space in the narrow vascular lumen, which can potentially confound pressure measurements hence contributing to the detection of falsely elevated gradients.

Despite being a strongly reliable and reproducible diagnostic modality, there are certain limitations of the FFR.

1- FFR requires drug administration, which can cause potential adverse effects including bradycardia, hypotension, and dyspnea causing significant patient discomfort [6].

2- FFR is an invasive, time-consuming and relatively expensive procedure [6].

3- Safety profile of FFR has significantly improved over time with better equipment and improved interventional techniques but still requires meticulous maneuvering of the equipment and can rarely cause vessel injury and dissection, warranting further interventions.

4- Performing FFR study in a patient with tortuous vascular anatomy, serial lesions, and severe calcifications can be particularly challenging [7].

PFFR correlation with ankle-brachial index

Unlike CFFR, there has been debate regarding the effectiveness and utility of PFFR in peripheral artery interventions.

Banerjee et al. evaluated the relationship of walking impairment, ankle-brachial index (ABI) and PFFR in patients with isolated superficial femoral artery (SFA) disease with no inflow or outflow lesions [8]. This was the first study conducted to evaluate the vitality of PFFR in peripheral interventions. The authors employed adenosine (100-200 µg) and nitroglycerine (100-200 µg) to induce hyperemia, and PFFR was measured using 0.014-inch Certus pressure wire. This study showed a strong correlation between walking impairment, PFFR and rest and exercise ABI.

Subsequently, Hioki et al. conducted a prospective study to evaluate the clinical utility of PFFR in patients with isolated iliac artery stenosis and correlated this finding with the post-exercise ABI [9]. A total of 16 patients were included in this study. The purpose of this study was to compare the hyperemic PFFR with rest and post-exercise ABIs with the severity of the lesion confirmed by Doppler ultrasound. The authors used papaverine at variable doses in the initial cohort of patients. They then used 20 mg as the optimal stable dose in the remainder of the patients, as there was no significant difference in PFFR at different doses. PFFR was measured using 0.014-inch (St. Jude Medical, Saint Paul, MN, USA) pressure wire. The authors found a significant correlation between PFFR and post-exercise ABI. There was also a significant improvement in PFFR after EVT.

Moreover, in this study, the post-exercise ABI was measured after a constant load, nonstandard protocol (2.4 km/h with a fixed grade of 12%). Various treadmill protocols affect the reliability and potentially influence the accuracy of post-exercise ABIs. Many treadmill protocols have been studied, but the most reliable protocol appears to be the “graded load test” (defined as the fixed speed with gradually increasing grade) rather than “constant load test” (defined as the fixed speed with fixed grade) [10]. As Hioki et al. used the “constant load test”, this might have influenced the reliability of the correlation between PFFR and post-exercise ABI. Another limitation of this particular study was its small sample size.

PFFR correlation with Doppler ultrasound

Lotfi et al. demonstrated the correlation of PFFR with peak systolic velocity (PSV) and the risk of restenosis [11]. This was a prospective, single-center study, which enrolled 20 patients with baseline ABIs and Doppler ultrasonography of the lower extremities. The study investigators administered adenosine 1 mg/kg to induce hyperemia and PFFR measured using a 0.014-inch (St. Jude Medical) pressure wire. Patients were serially followed with three ABIs and Doppler ultrasound readings of the involved extremities during the 1st year of follow-up.

The mean hyperemic PFFR before the intervention was 0.71 ± 3. Subsequently, the post-procedural hyperemic PFFR was dichotomized into two groups with PFFR >0.95 and <0.95. The patients with PFFR >0.95 demonstrated no significant increase in PSV over time. However, a PFFR <0.95 demonstrated a significant increase in PSV over time. Both groups demonstrated a similar rate of ipsilateral pain. Moreover, stent length and diameter did not change the PSV and ABI over time.

This study successfully established the correlation of PFFR with Doppler-measured PSV with some limitations. The study used an arbitrary cut off point for PSV and PFFR to identify the endpoint. Moreover, this study also used a liberal p-value of ≤0.15 as a significant value, which increases the likelihood of type 1 error.

A similar study was subsequently published that demonstrated the correlation of PFFR with peak systolic velocity ratio (PSVR) in iliofemoral atherosclerotic disease [12]. The authors administered isosorbide dinitrate 250 µg to induce hyperemia and PFFR measured using 0.014-inch (Prime Wire Prestige) pressure wire. The mean diameter of stenosis was 57.3% ± 13.2% and 23/40 lesions had intermediate (<75%) stenosis on angiography. Interestingly, the authors found that PSVR significantly correlated with PFFR with a sensitivity of 94% and a specificity of 50%. The cutoff value for a significant PFFR was 0.85 and for PSVR >2.5. Patients in the group had diffuse long lesions, thus possibly contributing to an inappropriate correlation of the pressure measurement during angiography to the position of the lesion evaluated on Doppler. This may have led to the poor specificity of PFFR in this study.

A study by Ruzsa et al. included patients with below knee artery (BKA) stenosis to evaluate the correlation of PFFR with noninvasive functional parameters before and after angioplasty [13]. This particular study included patients with angiographically significant lesions of BKA (diameter stenosis ≥70%). The investigators administered papaverine 40 mg to induce hyperemia and PFFR measured using the 0.014-inch Certus Pressure Wire (St. Jude Medical). The results validated a strong correlation of PFFR with Doppler measurement at stress, but no association at rest. The PFFR was also associated with percentage area stenosis and percentage diameter stenosis, but not with lesion length. Moreover, this study also demonstrated the correlation of PFFR with toe brachial index.

Given that there is a strong correlation of PFFR with stress Doppler ultrasound, yet making PFFR as a benchmark tool for endovascular treatments in clinical practice can be an overly simplistic assumption. More importantly, is the understanding of a physiological response to hyperemic conditions in peripheral artery disease [14]. Longstanding stenosis of the superficial femoral artery may lead to increased microvascular resistance after regulatory mechanisms are exhausted and therefore this may lead to decreased blood supply relative to the tissue demand.

Ikeoka et al. compared high hyperemic microvascular resistance (h-MR) with low h-MR before and after endovascular treatments of SFA [14]. The authors administered papaverine 20 mg to induce hyperemia and PFFR was measured using 0.014-inch Pressure/Doppler sensor wire (Combo Wire). There was a significant improvement in high h-MR after endovascular treatment compared to low h-MR values. But this correlation of h-MR was not significant with PFFR. It means there was no difference in distal pressure in SFA before and after hyperemia. The potential reason why the difference in h-MR and PFFR was not established is, that the flow in peripheral circulation is active velocity dependent while flow in coronary is passive pressure dependent. Due to a limited understanding of this pathophysiological mechanism, there is limited understanding of the use of PFFR. Moreover, there are limited studies done to explain the correlation of PFFR with Doppler.

PFFR in mesenteric ischemia

While there have been no retrospective or prospective studies reported to assess the role of PFFR in mesenteric ischemia, case reports have been published.

Sadiq et al. reported on a patient presenting with epigastric discomfort with eating, nausea, vomiting, weight loss, and epigastric bruit [15]. A magnetic resonance angiography (MRA) was suggestive of celiac artery stenosis. Mesenteric Doppler interrogation showed no significant obstruction, with a velocity of 100 cm/s during inspiration and 218 cm/s during expiration. Given a high clinical index of suspicion, the patient underwent a peripheral mesenteric angiogram, with PFFR measurement using a 0.014” 175 cm St Jude pressure wire which revealed severe stenosis of the proximal celiac artery during inspiration and expiration. Moreover, a PFFR during inspiration was 0.94 and 0.85 at baseline and hyperemia which was induced by intra-arterial administration of 300 μg nitroglycerin, respectively. During expiration, it was 0.84 and 0.78 at baseline and hyperemia, respectively. The PFFR value correlated with the MRA finding of dynamic celiac artery obstruction. In this case, PFFR helped in identifying the hemodynamic significance of the mesenteric artery lesion that had non-significant findings on Doppler.

Sadiq et al. reported on a 67-year-old male, presented with acute myocardial infarction, requiring emergent coronary artery bypass graft (CABG) surgery due to severe multi-vessel coronary artery disease [16]. Due to disabling symptoms secondary to severe obstructive disease of the distal abdominal aorta and bilateral common iliac arteries, the patient underwent a peripheral intervention. For that significant PAD, the patient had an aortic endograph that caused the occlusion of an already severe ostial stenosis of her inferior mesenteric. Four weeks after this intervention, the patient presented with postprandial abdominal pain. Computed tomography (CT) abdominal angiography demonstrated complete occlusion of the inferior mesenteric artery (IMA) with no significant stenosis of the celiac artery (CA) and superior mesenteric artery (SMA). Due to concerning clinical features, the patient underwent mesenteric angiography and demonstrated mild to moderate CA and moderate SMA disease with robust collaterals from SMA to IMA. The hemodynamic significance of CA and SMA was assessed sequentially by a 0.014” 175 cm Saint Jude pressure wire. A baseline PFFR of 0.87 followed by hyperemic PFFR of 0.65 recorded in SMA achieved by intra-arterial ingestion of 300 µg of nitroglycerin. The patient underwent stenting of SMA and post-procedure rest and hyperemic PFFR improved to 0.95 and 0.85, respectively. Similarly, baseline PFFR of 0.82 followed by hyperemic FFR of 0.75 was recorded in CA. The patient underwent stenting of the CA and post-procedure rest and hyperemic FFR improved to 0.97 and 0.82, respectively. Use of PFFR helped in hemodynamic assessment of mild to moderate angiographic narrowing of the arteries.

Invasive PFFR correlation with noninvasive PFFR

A small retrospective study was conducted on CT FFR in aortoiliac lesions [17]. In this study, all patients who had aortoiliac disease on CT scan underwent angiography. PFFR was measured angiographically using a 0.035 catheter in a retrograde fashion. It was found to have high sensitivity and specificity between measured PFFR by angiography and CT PFFR (100% sensitivity and specificity, with P = 0.986, area under the curve = 1).

The difference between measured PFFR by angiography and CT FFR was 0.136 (average: 0.03-0.30). CT PFFR successfully identified hemodynamically significant aortoiliac lesions with comparable results to invasive PFFR and it may have the potential to replace invasive PFFR.

Prediction of PFFR for future re-stenosis

Restenosis is one of the main concerns after endovascular treatment of peripheral artery disease. Previous studies reported the restenosis rate of superficial femoral artery 31%-63% after one year of EVT (nitinol stenting or angioplasty). Restenosis was assessed by Doppler US, computed tomographic angiography (CTA), invasive angiography and clinical outcomes [18-20].

Although patients overall medical history and risk factors profile, lesion severity and length, type of EVT, and interventional results are associated with future risk of restenosis. CFFR for future restenosis has been well studied. Pijls et al. demonstrated the association of post-intervention CFFR was inversely associated with a major cardiovascular event and future risk of restenosis [21]. Little is known about PFFR and future risk of restenosis. The first study was conducted by Lotfi et al. regarding PFFR and future risk of peripheral restenosis [11]. A post-intervention PFFR of <0.95 was associated with a significant increase in PSV. Later on, Kobayashi et al. conducted a single center nonrandomized prospective study to demonstrate the relation of post-intervention PFFR and future risk of restenosis [22]. The authors administered papaverine 30 mg to induce hyperemia and PFFR was measured using 0.014-inch Pressure Wire Aeris G8 (St. Jude Medical). In this study, the authors described post-intervention PFFR as a mean and systolic PFFR. However both post-intervention PFFR were significantly lower in restenosis group (mean PFFR: 0.85 ± 0.07, systolic PFFR: 0.76 ± 0.14) than no restenosis group (mean PFFR: 0.93 ± 0.05, systolic PFFR: 0.87 ± 0.08). However, there was no statistically significant difference between mean PFFR and systolic PFFR. Unlike the previous study where the cut off value of post-intervention PFFR was 0.95, in this study the cut off value for post-intervention mean PFFR was 0.92 with sensitivity and specificity of 64% and 91%, respectively. The post-intervention mean PFFR ≤0.92 had a restenosis rate of 35.7% compared to 4.5% in post-intervention mean PFFR > 0.92.

The differences of cut off value between two studies were likely due to:

1- Lotfi et al. used fixed adenosine dose 1 mg/kg bolus, so whether the dose was sufficient or not is unknown. So if the adenosine dose was insufficient to produce maximal hyperemia, then PFFR would be underestimated.

2- In the periphery, the sheath location is important because the sheath is able to decrease peripheral blood flow by occupying the lumen, which may also lead to underestimating the PFFR [23].

## Conclusions

Given the limited evidence-based research to date, the clinical efficacy of the use of FFR in peripheral intervention is still a question. The correlation of PFFR with ABI and Doppler ultrasound is seen in limited and observational studies. Unlike CFFR, there are no prospective randomized controlled trials to ascertain the correlation of PFFR with ABI and Doppler ultrasound. The optimal agents and doses of the hyperemic agents are well established in CFFR. However, the optimal agents and doses of the hyperemic agents are not well established in PFFR yet. Moreover, unlike CFFR, there is currently no well-established reliable protocol to measure PFFR.

Further studies are needed to evaluate the need of the PFFR along with the establishment of standardized procedural protocols to measure the PFFR for this powerful diagnostic tool to be reliably utilized in the management of peripheral artery disease.
